# Supplementary material for: New model of glucose-insulin regulation characterizes effects of physical activity and facilitates personalized treatment evaluation in children and adults with type 1 diabetes
Source: PLoS Comput Biol. 2023 Feb 15;19(2):e1010289. doi: 10.1371/journal.pcbi.1010289 (PMC9974135; doi:10.1371/journal.pcbi.1010289)
Supplement: S1 File — Definition of standard patient. Additional information on replay simulations. (PDF) [file pcbi.1010289.s001.pdf]

Supplementary Material

New model of glucose-insulin regulation characterizes effects of physical activity and facilitates personalized treatment evaluation in children and adults with type 1 diabetes

Julia Deichmann, Sara Bachmann, Marie-Anne Burckhardt,
Marc Pfister, Gabor Szinnai, Hans-Michael Kaltenbach

Section 1 Calibration of Exercise Model

Section 1.1 Parameter Determination for Healthy Subjects

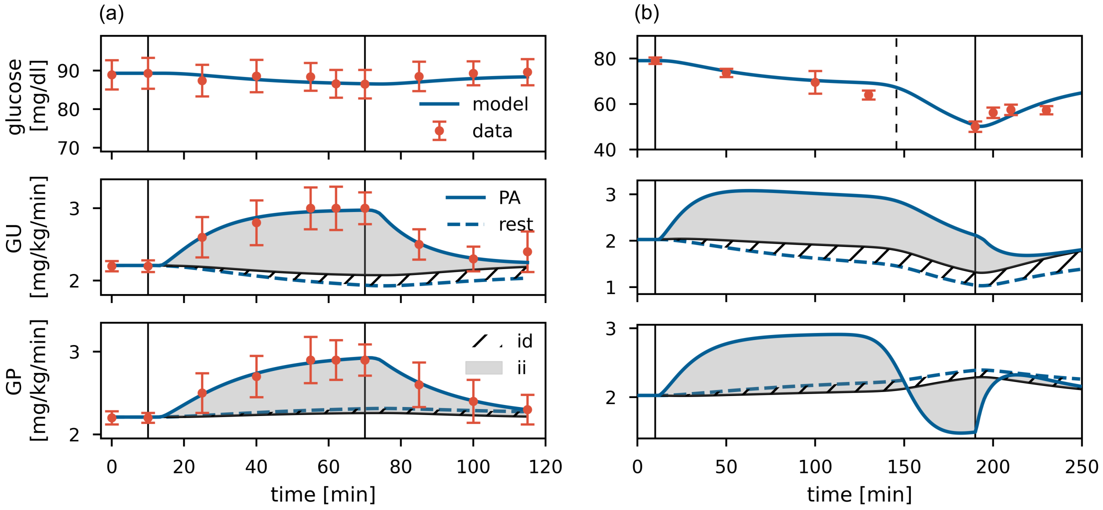

Fig A. (a) Data (mean ± SEM, n = 8) [1] and model fit for 60 min of PA at 40% VO_2^{max}. (b) Data (mean ± SEM, n = 10) [2] and model fit for 180 min of PA at 58% VO_2^{max}. Glucose levels and GU and GP rates are shown. The PA session is marked by solid lines and onset of depletion by dashed lines. PA-driven changes in GU and GP are separated into insulin-dependent (id) and insulin-independent (ii) contributions.

Table A. Model parameters. Healthy: healthy subjects. V1–V3: T1D subjects under euglycemia low-insulin (V1), euglycemia high-insulin (V2), and hyperglycemia low-insulin (V3) clamp conditions [3, 4].

Parameter	Healthy	T1D			Unit
		V1	V2	V3	
**Glucose-Insulin Regulation**					
p_1	0.017	0.0126	0.0214	0.0114	1/min
p_2	0.0351	0.0228	0.0228	0.0228	1/min
p_3	$2.47 \cdot 10^{-5}$	$2.78 \cdot 10^{-5}$	$2.98 \cdot 10^{-5}$	$2.19 \cdot 10^{-5}$	ml/(μU·min^2)
p_4	0.058	0.058	0.058	0.058	1/min
p_5	0.0885	0.0885	0.0885	0.0885	1/min
V_g	1.289	1.289	1.289	1.289	dl/kg
**Insulin Sensitivity**					
τ_{AC}	5	5	5	5	min
τ_Z	600	600	600	600	min
b	$1.68 \cdot 10^{-6}$	$3.64 \cdot 10^{-6}$	$1.59 \cdot 10^{-6}$	$1.55 \cdot 10^{-6}$	1/count
**Glucose Uptake and Production**					
α	0.27	0.27	0.27	0.27	dimensionless
q_1	$1.93 \cdot 10^{-7}$	$6.46 \cdot 10^{-7}$	$2.88 \cdot 10^{-6}$	$5.23 \cdot 10^{-7}$	1/(count·min)
q_2	0.066	0.0617	0.2682	0.0778	1/min
q_{3l}	$1.15 \cdot 10^{-7}$	$4.46 \cdot 10^{-7}$	$1.33 \cdot 10^{-7}$	$1.60 \cdot 10^{-7}$	1/(count·min)
q_{4l}	0.051	0.0705	0.0299	0.0669	1/min
q_{3h}	-	-	-	$5.87 \cdot 10^{-7}$	1/(count·min)
q_{4h}	-	-	-	0.056	1/min
q_5	-	-	-	0.03	1/min
**Glycogen Depletion**					
β	0.76	-	-	-	dimensionless
q_6	0.1	-	-	-	1/min
a_{depl}	0.0108	-	-	-	min^2/count
b_{depl}	180.6	-	-	-	min
**Transfer Functions**					
a_Y	1500	1500	1500	1500	counts/min
a_{AC}	1000	1000	1000	1000	counts/min
a_h	5600	5600	5600	5600	counts/min
t_p	2	2	2	2	min
n_1	20	20	20	20	dimensionless
n_2	100	100	100	100	dimensionless

Section 1.2 Adjustment of Model Parameters to T1D

Section 1.2.1 Parameter Estimation Strategy

We used data by Romeres et al. [3, 4] to re-calibrate the exercise model to people with T1D. GU and GP were studied during 60 min of exercise at 65% VO_2^{max} under three glucose and insulin conditions (V1: euglycemia – low insulin, V2: euglycemia – high insulin, V3: hyperglycemia – low insulin). When studying glucose uptake, Romeres et al. found that parameter p_2 and the glucose distribution volume V_g were similar across all study conditions, while the participants' insulin sensitivity (SI, corresponding to p_3) and glucose effectiveness p_1 varied. Hence, we fixed V_g and p_2 (p_2 determined from GIR required after insulin aspart injection [5]) as well as the exchange rates p_4 and p_5 (since glucose fluctuations are small), and only estimated parameters p_1 and p_3 of the glucose-insulin model. In addition, we estimated exercise parameters b, q_1, q_2, q_{3l} and q_{4l} as well as basal levels of glucose, G_b, and insulin, I_b. We fixed $\tau_Z = 600\,\text{min}$, which defines the return of insulin sensitivity to its baseline, since it is difficult to estimate such a time scale correctly based on the comparatively short recovery period included in the data.

Our parameter estimation strategy entails two steps. First, we identified parameters for condition V1 from GU and GP data and used glucose and insulin measurements as known inputs. This condition represents the physiologically 'normal' scenario and hence, we used the resulting parameters as our baseline description of T1D populations for the remainder of this work. Second, we tested whether our model is applicable also to other glucose and insulin scenarios, and estimated parameters for conditions V2 and V3. We used the V1 parameter estimates as prior information to constrain the parameter estimation for these conditions, since they share the same study population.

Condition V1: euglycemia - low insulin We determined parameters for condition V1 using maximum likelihood estimation, minimizing the negative log-likelihood

$$-2\,LL(y|\theta) = \sum_{i=1}^{n} \frac{(y_i - f(t_i,\theta))^2}{\sigma^2},$$

which corresponds to a Gaussian distribution of data points y_i around model predictions $f(t_i, \theta)$ at time t_i with parameter set θ and residual variance σ^2. For the minimization, we used the Nelder-Mead algorithm in Python's scipy.optimize package.

Parameters p_1 and p_3 are not identifiable in the unconstrained optimization problem (see below). However, insulin-independent and -dependent contributions to GU are similar at rest in the original study and we exploited this fact by enforcing the relation $p_1 = X_b = p_3/p_2 \cdot I_b$ during optimization, which makes the parameters identifiable. We also detected an outlier in GU at $t = 190\,\text{min}$, which we removed to improve estimation.

Conditions V2: euglycemia – high insulin, and V3: hyperglycemia – low insulin We used the parameter estimates θ_j^{V1} from condition V1 to constrain the estimation problem for conditions V2 and V3. For this, we added a penalty term to the log-likelihood function that corresponds to a log-normal distribution of parameter values around the $V1$ estimates with about 60% relative standard deviation. The penalized log-likelihood for the optimization is then

$$-2\,LL(y|\theta) = \sum_{i=1}^{n} \frac{(y_i - f(t_i,\theta))^2}{(\sigma^{V1})^2} + \lambda \cdot \sum_{j=1}^{k} \frac{\left((ln(\theta_j) - ln(\theta_j^{V1})\right)^2}{ln(1.6)^2}.$$

We used the residual standard deviation σ^{V1} from the previous estimate. We did not employ the previous constraint on p_1 and p_3, which are freely estimated during this optimization.

The resulting glucose production and uptake rates are shown in Figure B. For all conditions, the model captures the exercise-driven changes in GU and GP well. Parameter values are reported in Table A and our results agree qualitatively with the assessment of exercise effects in the original study, also in terms of the differences observed between conditions.

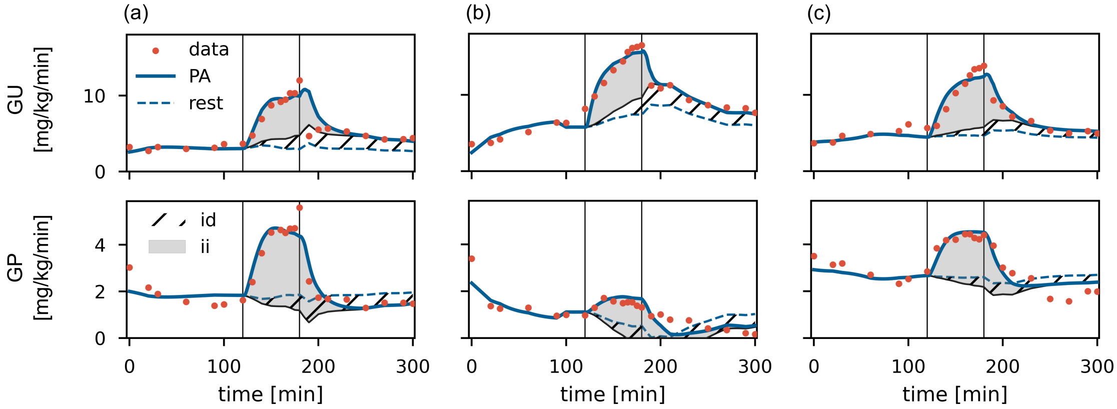

Fig B. Data (mean, n = 6) [3, 4] and model fits of GU and GP rates during 60 min of PA at 65% VO_2^{max} under (a) euglycemia - low insulin (V1), (b) euglycemia - high insulin (V2), and (c) hyperglycemia - low insulin (V3) conditions. The PA session is marked by vertical lines. PA-driven changes in GU and GP are separated into insulin-dependent (id) and insulin-independent (ii) contributions. Note that GU and GP rates are always positive, while exercise-induced changes can have a negative effect.

Section 1.2.2 Parameter Identifiability

Next, we considered practical identifiability of parameters and computed profile likelihoods (PL) to obtain appropriate confidence intervals (CI) [6]. The profile likelihood is the log-likelihood function evaluated for a specific parameter θ_j with values p and maximized over all remaining parameters:

$$PL_j(p) = \max_{\theta \in \{\theta | \theta_j = p\}} LL(y|\theta)$$

The $(1-\alpha)$ confidence interval for θ_j is then found from the quantiles of the χ^2-distribution with one degree of freedom.

Based on the available data, some parameters are not identifiable for condition V1 (Fig. C (a)). Glucose and insulin levels are constant during the study period, impeding for example the separation of insulin-dependent and –independent processes already at rest. The parameter correlations (Fig. C (b)) reveal more insights: p_1 and p_3 correlate negatively under condition V1 and can compensate for each other, confirming the observed identifiability issues. The exercise parameters q_1 and q_2, and similarly q_3 and q_4, also show strong correlations. This is expected, since the steady state values of the GU and GP rates can be maintained when the ratio of the two corresponding parameters is held constant. The profile likelihoods for conditions V2 and V3 (Fig. C (c),(d)) show more confined parameter ranges. This is due to changes in glucose or insulin concentration during the study, allowing to distinguish between insulin-dependent and -independent contributions, and was further improved by the penalty term used during optimization.

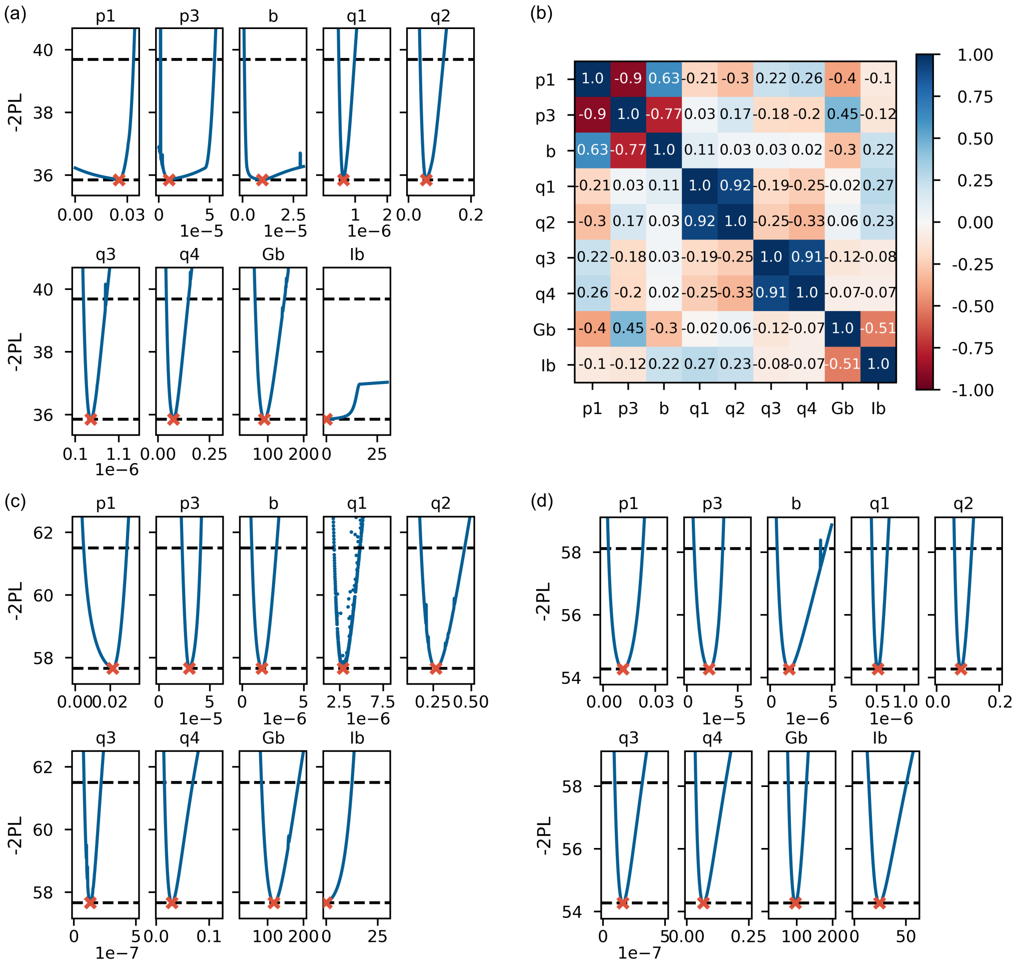

Fig C. (a) Profile likelihoods and (b) parameter correlations for condition V1. Profile likelihoods for conditions (c) V2 and (d) V3. 95% confidence thresholds enclosing the parameters' confidence intervals are marked by the upper dashed lines.

Section 1.3 High-Intensity Exercise

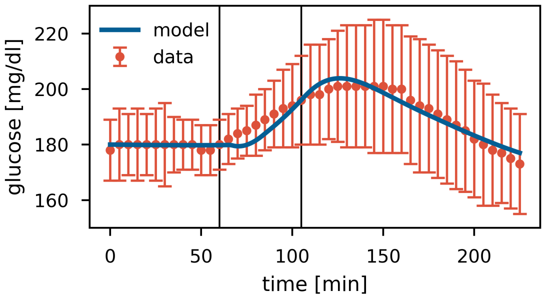

Fig D. Data (mean ± SEM, n = 12) [7] and model fit of glucose levels during 45 min of PA at 82.5% VO_2^{max}. The high-intensity PA session is marked by vertical lines.

Section 2 Parameters of Insulin Kinetics Model

We used data from a published study on insulin kinetics to estimate the parameters of the insulin input component [8]. Parameter values are given in Table B, and provide an excellent fit to the published data (Fig. E).

Table B. Estimated parameters of the insulin kinetics model.

Parameter	Value
k_1	0.0085
k_2	0.011
k_3	0.0247
k_4	0.0357
V_I	104

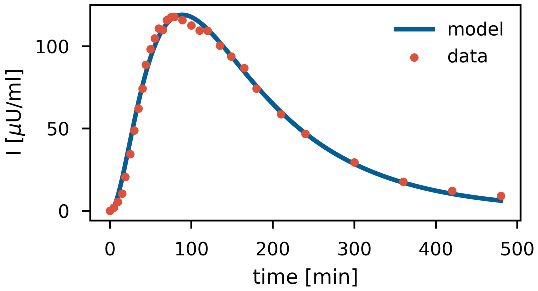

Fig E. Data [8] and model fit of plasma insulin after injection of 0.3U/kg insulin aspart.

Section 3 Model Validation

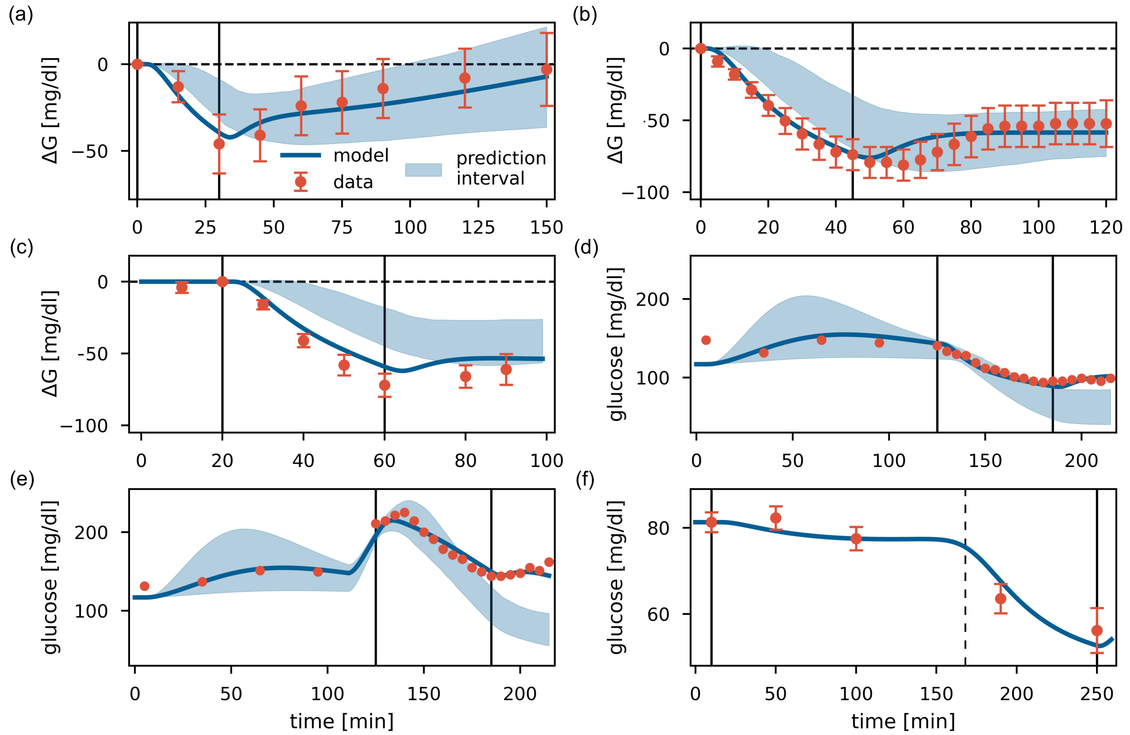

Fig F. Data (mean ± SEM) and model predictions for model validation. (a)-(e) Shaded areas show the 95% prediction intervals of the T1D model (V1), and solid BG trajectories display the tuned model. (a) Validation study 2 (n = 8; $b = 5.97 \cdot 10^{-6}$, $q_1 = 2.69 \cdot 10^{-6}$, $q_2 = 0.2569$) [9], (b) validation study 3 (n = 11; $b = 4.84 \cdot 10^{-6}$, $q_1 = 2.06 \cdot 10^{-6}$, $q_2 = 0.1968$) [10] and (c) validation study 4 (n = 12; $b = 9.10 \cdot 10^{-6}$, $q_1 = 3.23 \cdot 10^{-6}$, $q_2 = 0.3085$) [11]. Validation study 5 [12] (d) without pre-PA snack and (e) with pre-PA snack (n = 11; $p_3 = 1.15 \cdot 10^{-5}$, $b = 1.30 \cdot 10^{-6}$, $q_1 = 2.54 \cdot 10^{-6}$, $q_2 = 0.2426$). Meal parameters are $f = 0.55$ and $\tau_m = 90\,\text{min}$ for lunch and $f = 1.8$ and $\tau_m = 20\,\text{min}$ for the pre-PA CHOs. (f) Validation study 6 [13] with parameters determined for healthy subjects. The PA sessions are marked by solid vertical lines and onset of depletion by dashed vertical lines.

Section 4 Definition of Standard Patient

For our full-day simulation results, we defined a standard patient with parameter values taken over from the model calibration for T1D (condition V1). However, data did not include glycogen depletion and high intensity for this condition, and we therefore used depletion parameters β and q_6 from healthy subjects, assuming that the contribution of glycogenolysis to overall GP during PA is similar for healthy and T1D subjects. In condition V3, GP exceeds GU during high-intensity PA by a factor of 1.6 at steady state. We assumed the same ratio here to determine high-intensity parameters q_{3h} and q_{4h}.

Section 5 Replay Simulations

Section 5.1 In-Silico Performance Assessment of Model Personalization

We tested the performance of the personalized model in replay simulations in-silico, re-creating the experiments of the original study [14]. We used the UVa/Padova simulator [15] (Python implementation [16]) to generate individual data of 10 virtual subjects. We then personalized our model on these data and performed replay simulations with altered meal and insulin bolus inputs. We followed Hughes et al. [14] and evaluated the replay performance using the mean absolute relative difference (MARD) between 'true' and replayed glucose trajectories.

Specifically, each individual received a meal containing 0.7gCHO/kg at 12:00 in a 24h simulation using the UVa/Padova simulator. A meal bolus was injected at the same time, where the bolus dose was computed according to a personal insulin-to-carbohydrate ratio. We personalized the model on these data to each subject. We fixed the parameters at their T1D population values and only estimated p_1, p_3, G_b, f and τ_m, with τ_m constrained between 10 and 150 min.

In the first experiment, we changed the meal size from 0 to 200% of the original meal size in steps of 20%. For each subject, we simulated 'true' data with the UVa/Padova simulator, and we simulated replay predictions according to our personalized models. The resulting MARD between 'true' and replayed data starting from meal time at 12:00 is shown in Fig. G (a).

To evaluate the impact of insulin inputs, we varied the insulin bolus from 50 to 150% of the original bolus in steps of 10% in a second experiment (Fig. G (b)).

In both simulation experiments, we found an acceptable MARD of less than 10% in most cases, but exceeded this threshold slightly for more extreme alterations. Note that in contrast to the replay simulations shown in Hughes et al. [14], we did not consider further deconvolution to feed unaccounted dynamics as additional inputs into the simulation. Rather, we relied solely on adjusting model parameters to individual subjects, and accepted slightly higher MARD values in return.

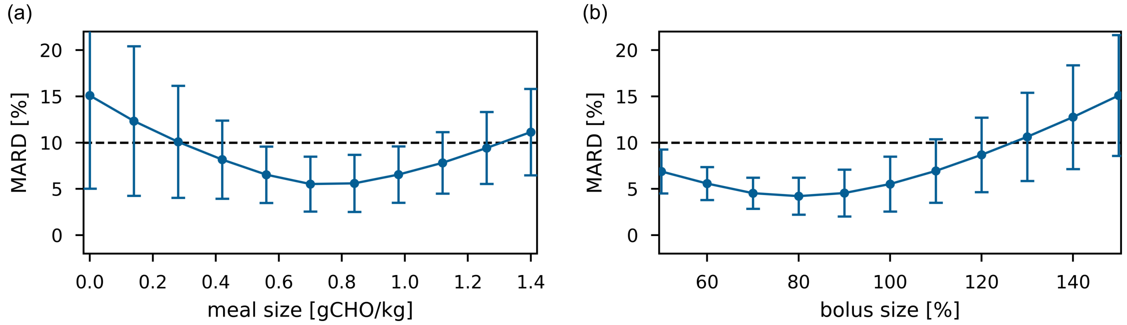

Fig G. Results of the replay simulations. MARD (mean and standard deviation) between true simulated data and replay predictions for alterations in (a) meal size and (b) insulin dose.

Section 5.2 Model Personalization on T1D Data

Section 5.2.1 Characteristics of Study Participants

We selected five pediatric T1D participants for model personalization. Baseline characteristics are presented in Table C.

Table C. Baseline characteristics of study participants.

Participant	f/m	age [y]	weight [kg]	insulin [U/kd/d]	% basal	treatment	u_b reported
# 1	f	8	34.7	0.9	59	MDI	n/a
# 2	m	13	58.8	0.9	54	CSII	no
# 3	m	14	56.3	0.8	45	CSII	no
# 4	m	10	31.6	0.8	52	MDI	n/a
# 5	m	10	38.8	0.6	48	CSII	yes

Section 5.2.2 Data Preparation

The recorded study data consist of glucose measurements, accelerometer counts, timing and dosing of insulin injections and timing and carbohydrate content of meals. Glucose data were recorded continuously by a CGM device. Continuous exercise data were obtained using an Actigraph model GT3X+ accelerometer (ActiGraph, LLC; Pensacola, Florida, USA) that is worn on the right hip. Acceleration is measured along three axes, and data of the vertical axis were used to quantify intensity of movement. In contrast, participants or their caregivers manually reported insulin injections and meals in logbooks. Discrepancies between the provided information and the measured glucose levels indicate partly inaccurate or incomplete logbook data. While incorrectly estimated meal sizes were compensated for by allowing arbitrary values of the bioavailability factor f in the meal model, we had to manually account for incorrect meal times and for missing meals. To this end, we shifted meal times and the corresponding insulin bolus times to adjacent glucose minima if required. On few occasions, we found small glucose peaks during the day without reported meals or other disturbances, and introduced additional snacks without insulin bolus in these cases. All meal adjustments are given in Table E.

To extract PA intervals, we smoothed the AC data using a median filter with a window size of 15 min. We identified intervals exceeding 1500 counts/min for at least 10 min as PA sessions, according to the threshold for minimum PA intensity set in the model. We merged sessions that were separated by less than 10 min into a single PA period.

Finally, we needed to define the basal insulin infusion rate u_b for each individual. For study participants on multiple daily injection (MDI) therapy, we assumed a constant basal rate, which we determined from the person's total daily insulin requirements and the contribution from basal injections. For participants on continuous subcutaneous insulin infusion (CSII) therapy, we either used the reported values for u_b, or applied the same strategy as for MDI therapy if u_b was not reported (Table C).

Section 5.2.3 Personalized Model

Parameter values of the personalized models for each of the five children are given in Tables D and E. The resulting model fits are shown in Figure 5 of the main text for two participants, and in Figure H for the remaining participants. Due to missing information, we were unable to fit a small number of glucose excursions either in the early morning or late evening; these are listed in Table F and were ignored for model personalization.

Table D. Personalized model parameters.

Parameter	Participant				
	# 1	# 2	# 3	# 4	# 5
p_1	$4.51 \cdot 10^{-11}$	$4.50 \cdot 10^{-4}$	$3.59 \cdot 10^{-8}$	0.0003	$5.53 \cdot 10^{-9}$
p_3	$0.62 \cdot 10^{-5}$	$1.48 \cdot 10^{-5}$	$1.65 \cdot 10^{-5}$	$1.49 \cdot 10^{-5}$	$2.45 \cdot 10^{-5}$
G_b, d1	167.7	60.7	149.6	176.9	94.0
G_b, d2	30.0	69.1	120.0	108.3	60.6

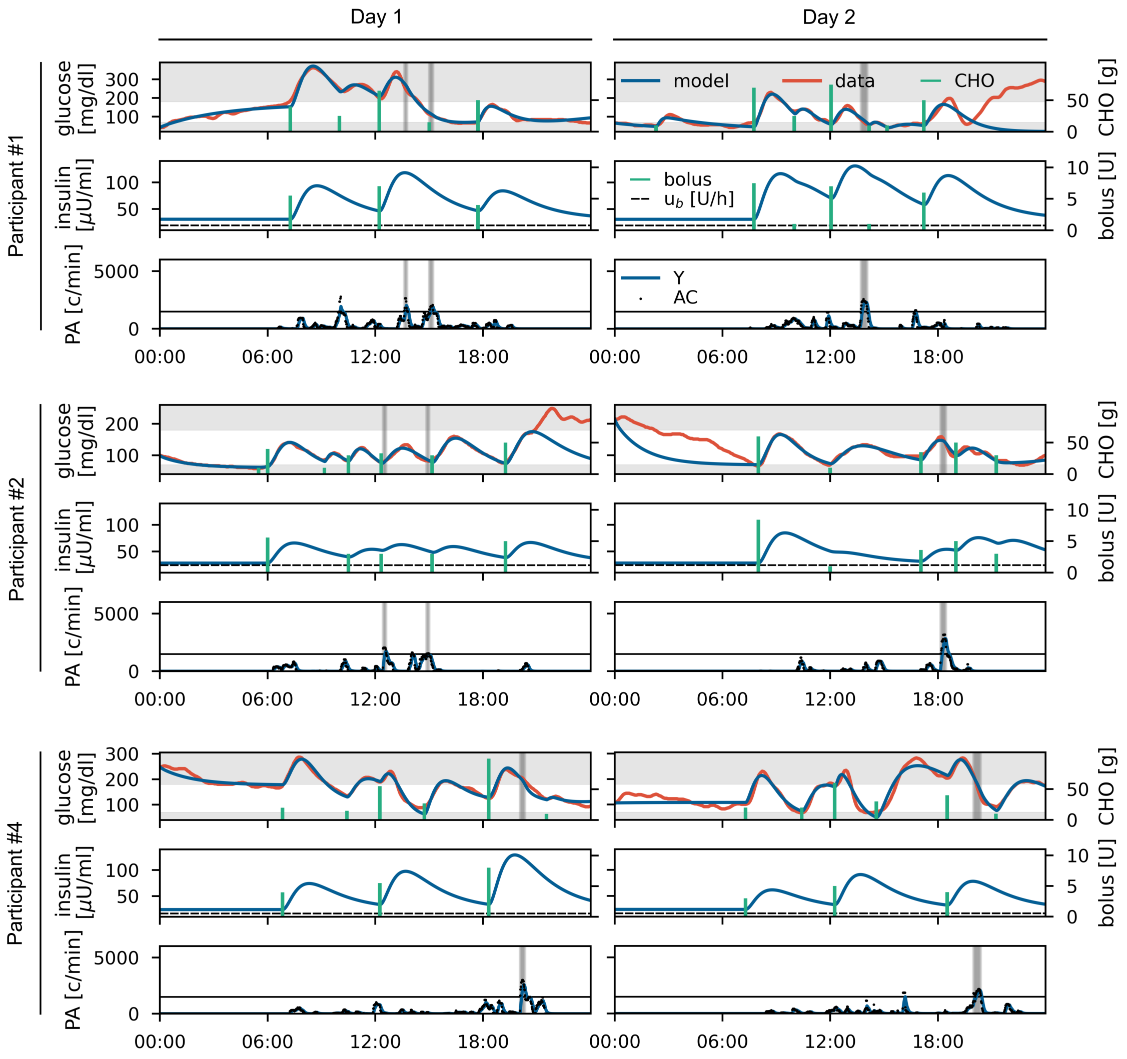

Fig H. Data and personalized model for participants #1, #2 and #4 for two days each. For each day, recorded (red) and fitted (blue) glucose data and carbohydrate inputs (green) are shown in the upper panel. Modelled insulin concentration (blue) and insulin inputs (green) including the basal insulin infusion rate (dashed) are shown in the middle panel. Accelerometer counts (dotted) and modelled PA intensity Y (blue) are shown in the lower panel with periods of physical activity highlighted in grey.

Table E. Meal information. Time (including shift from reported logbook data) and CHO amount. Parameters f and τ_m.

Participant	Day 1				Day 2			
	time	CHO [g]	f	τ_m [min]	time	CHO [g]	f	τ_m [min]
#1	07:16	40	0.94	36	02:18 (+0:10)	10	0.44	10
	10:00	25	0.70	25	07:45	70	0.35	25
	12:13	65	0.40	30	10:00	25	0.35	15
	15:00	15	0.00	10	12:03	75	0.21	22
	17:43	50	0.18	14	14:10 (+1:10)	10	0.40	10
	–	–	–	–	15:10 (+0:10)	10	0.83	35
	–	–	–	–	17:13 (−1:00)	50	0.48	32
#2	05:30	10	1.22	143	08:00	60	1.16	60
	06:00	40	0.91	48	12:00 (−0:30)	10	3.91	59
	09:10 (+)	10	0.79	12	17:03	35	0.80	37
	10:30 (+0:30)	30	0.45	18	19:00	50	0.83	49
	12:20 (+0:20)	33	1.19	44	21:15	30	2.56	150
	15:10 (−0:20)	30	1.83	49	–	–	–	–
	19:15 (−0:45)	50	1.70	66	–	–	–	–
#3	06:05	55	1.01	68	09:25 (+0:12)	65	0.54	43
	10:10	55	0.18	22	11:15 (+)	20	2.16	44
	12:20 (+0:15)	70	0.76	67	12:45 (+0:15)	35	1.45	63
	17:10 (+0:10)	35	0.13	18	16:15 (+0:11)	10	0.81	18
	19:30 (−0:30)	65	1.98	70	19:05 (−0:25)	35	0.18	24
	–	–	–	–	20:35 (−0:15)	50	2.02	80
	–	–	–	–	22:40	15	0.60	13
#4	06:50 (−0:17)	20	1.91	54	07:17	20	0.84	26
	10:25 (+0:25)	15	1.03	30	10:25 (+0:15)	20	1.04	33
	12:15	55	0.12	16	12:15	60	0.11	10
	14:44	27	1.28	33	14:34	30	2.48	64
	18:19	100	0.88	76	18:31 (+0:05)	40	0.51	31
	21:32 (+0:25)	10	0.20	10	21:14 (+0:20)	10	3.96	48
#5	00:43	30	0.92	59	07:55	20	0.87	16
	09:24 (+0:15)	30	1.32	47	09:30	50	1.45	55
	13:55 (+0:55)	70	0.15	14	13:14 (+0:07)	55	1.46	49
	15:30 (+)	30	0.77	26	17:24	30	2.90	53
	16:39 (+0:10)	30	5.16	67	20:11	50	0.92	76
	19:26 (−0:10)	30	0.00	150	–	–	–	–

Table F. Timing of glucose excursions discarded for model personalization.

Day	Participant				
	#1	#2	#3	#4	#5
1	–	from 20:30	–	–	–
2	from 20:00	until 8:00	–	–	until 6:40

References

1. Wolfe RR, Nadel ER, Shaw JHF, Stephenson LA. Role of changes in insulin and glucagon in glucose homeostasis in exercise. Journal of Clinical Investigation. 1986;77(3):900–907. doi:10.1172/JCI112388.

2. Ahlborg G, Felig P. Lactate and glucose exchange across the forearm, legs, and splanchnic bed during and after prolonged leg exercise. Journal of Clinical Investigation. 1982;69(1):45–54. doi:10.1172/JCI110440.

3. Romeres D, Schiavon M, Basu A, Cobelli C, Basu R, Dalla Man C. Exercise Effect on Insulin-Dependent and Insulin-Independent Glucose Utilization in Healthy and Type 1 Diabetes Individuals. A Modeling Study. American Journal of Physiology-Endocrinology and Metabolism. 2021;doi:10.1152/ajpendo.00084.2021.

4. Romeres D, Basu A, Schiavon M, Cobelli C, Man CD, Basu R. Effects of Hyperglycemia and Hyperinsulinemia on Glucose Turnover during Exercise in Type 1 Diabetes. Diabetes. 2018;67(Supplement 1).

5. Heise T, Stender-Petersen K, Hövelmann U, Jacobsen JB, Nosek L, Zijlstra E, et al. Pharmacokinetic and Pharmacodynamic Properties of Faster-Acting Insulin Aspart versus Insulin Aspart Across a Clinically Relevant Dose Range in Subjects with Type 1 Diabetes Mellitus. Clinical Pharmacokinetics. 2017;56(6):649–660. doi:10.1007/s40262-016-0473-5.

6. Kreutz C, Raue A, Kaschek D, Timmer J. Profile likelihood in systems biology. The FEBS Journal. 2013;280(11):2564–2571. doi:https://doi.org/10.1111/febs.12276.

7. Jayawardene DC, McAuley SA, Horsburgh JC, Gerche AL, Jenkins AJ, Ward GM, et al. Closed-loop insulin delivery for adults with type 1 diabetes undertaking high-intensity interval exercise versus moderate-intensity exercise: A randomized, crossover study. Diabetes Technology and Therapeutics. 2017;19(6):340–348. doi:10.1089/dia.2016.0461.

8. Svehlikova E, Mursic I, Augustin T, Magnes C, Gerring D, Jezek J, et al. Pharmacokinetics and pharmacodynamics of three different formulations of insulin aspart: A randomized, double-blind, crossover study in men with type 1 diabetes. Diabetes Care. 2021;44(2):448–455. doi:10.2337/dc20-1017.

9. Maran A, Pavan P, Bonsembiante B, Brugin E, Ermolao A, Avogaro A, et al. Continuous glucose monitoring reveals delayed nocturnal hypoglycemia after intermittent high-intensity exercise in nontrained patients with type 1 diabetes. Diabetes Technology and Therapeutics. 2010;12(10):763–768. doi:10.1089/dia.2010.0038.

10. Iscoe KE, Riddell MC. Continuous moderate-intensity exercise with or without intermittent high-intensity work: Effects on acute and late glycaemia in athletes with Type1 diabetes mellitus. Diabetic Medicine. 2011;28(7):824–832. doi:10.1111/j.1464-5491.2011.03274.x.

11. Zaharieva D, Yavelberg L, Jamnik V, Cinar A, Turksoy K, Riddell MC. The effects of basal insulin suspension at the start of exercise on blood glucose levels during continuous versus circuit-based exercise in individuals with type 1 diabetes on continuous subcutaneous insulin infusion. Diabetes Technology and Therapeutics. 2017;19(6):370–378. doi:10.1089/dia.2017.0010.

12. Dubé MC, Lavoie C, John Weisnagel S. Glucose or intermittent high-intensity exercise in glargine/glulisine users with T1DM. Medicine and Science in Sports and Exercise. 2013;45(1):3–7. doi:10.1249/MSS.0b013e31826c6ad3.

13. Ahlborg G, Felig P, Hagenfeldt L, Hendler R, Wahren J. Substrate turnover during prolonged exercise in man. Splanchnic and leg metabolism of glucose, free fatty acids, and amino acids. Journal of Clinical Investigation. 1974;53(4):1080–1090. doi:10.1172/JCI107645.

14. Hughes J, Gautier T, Colmegna P, Fabris C, Breton MD. Replay Simulations with Personalized Metabolic Model for Treatment Design and Evaluation in Type 1 Diabetes. Journal of Diabetes Science and Technology. 2021;15(6):1326–1336. doi:10.1177/1932296820973193.

15. Dalla Man C, Micheletto F, Lv D, Breton M, Kovatchev B, Cobelli C. The UVA/PADOVA type 1 diabetes simulator: New features. Journal of Diabetes Science and Technology. 2014;8(1):26–34. doi:10.1177/1932296813514502.

16. Xie J. Simglucose v0.2.1; 2018. Available from: https://github.com/jxx123/simglucose.
